# Lung Injury Prediction Score Is Useful in Predicting Acute Respiratory Distress Syndrome and Mortality in Surgical Critical Care Patients

**DOI:** 10.1155/2015/157408

**Published:** 2015-08-02

**Authors:** Zachary M. Bauman, Marika Y. Gassner, Megan A. Coughlin, Meredith Mahan, Jill Watras

**Affiliations:** Henry Ford Hospital, Detroit, MI 48202, USA

## Abstract

*Background*. Lung injury prediction score (LIPS) is valuable for early recognition of ventilated patients at high risk for developing acute respiratory distress syndrome (ARDS). This study analyzes the value of LIPS in predicting ARDS and mortality among ventilated surgical patients.* Methods*. IRB approved, prospective observational study including all ventilated patients admitted to the surgical intensive care unit at a single tertiary center over 6 months. ARDS was defined using the Berlin criteria. LIPS were calculated for all patients and analyzed. Logistic regression models evaluated the ability of LIPS to predict development of ARDS and mortality. A receiver operator characteristic (ROC) curve demonstrated the optimal LIPS value to statistically predict development of ARDS.* Results*. 268 ventilated patients were observed; 141 developed ARDS and 127 did not. The average LIPS for patients who developed ARDS was 8.8 ± 2.8 versus 5.4 ± 2.8 for those who did not (*p* < 0.001). An ROC area under the curve of 0.79 demonstrates LIPS is statistically powerful for predicting ARDS development. Furthermore, for every 1-unit increase in LIPS, the odds of developing ARDS increase by 1.50 (*p* < 0.001) and odds of ICU mortality increase by 1.22 (*p* < 0.001).* Conclusion*. LIPS is reliable for predicting development of ARDS and predicting mortality in critically ill surgical patients.

## 1. Introduction

Mechanical ventilation is one of the most common treatment modalities utilized in the intensive care unit (ICU) regardless of the admission reason [1]. Up to 25% of patients with normal lungs will develop some level of acute respiratory distress syndrome (ARDS) when placed on mechanical ventilation [2]. Much of this lung injury can be attributed to the use of higher tidal volumes [1, 3] as demonstrated by work done by the Acute Respiratory Distress Syndrome Network in 2000 [4]. Furthermore, surgical patients in the ICU are a unique subset of critical patients as they frequently experience physiological stresses directly related to surgery or trauma resulting in a higher likelihood of developing ARDS [5]. ARDS has been identified as the most common cause of postoperative respiratory failure [6] with high morbidity and mortality despite implementation of preventative strategies.

ARDS is theorized to result from injury to the alveolar epithelium and capillary endothelium with alterations in the immune system [7]. Neutrophils are recruited to the lungs by cytokines, become activated, and release toxic mediators, such as reactive oxygen species and proteases [8]. Extensive free radical production overwhelms endogenous antioxidants and causes oxidative cell damage [8]. Factors such as endothelin-1, angiotensin-2, NF-kappa B, and phospholipase A-2 increase vascular permeability and destroy microvascular architecture, enhancing inflammation and lung damage [8]. This results in an influx of protein-rich fluid into the airspaces from an increased permeability of the alveolar-capillary barrier [7]. The increase of alveolar fluid decreases gas exchange across the alveolar-capillary membrane resulting in hypoxemia and respiratory failure. This further leads to generation of reactive oxygen species [9] and activation of the coagulation cascade [10] promoting additional lung injury. It has been shown that various surgical insults and perioperative healthcare delivery factors such as blood product transfusions, intravenous fluid administration, and ventilator management strategies can impact these lung injury pathways [5].

Current management strategies for ARDS are multiple and often multifactorial. Most commonly, physicians will attempt a low tidal volume strategy (tidal volume of 6–8 mL/kg ideal body weight) based on the Acute Respiratory Distress Syndrome Network study in 2000 [4]; however this is not without its own complications which can include hypercapnic respiratory acidosis [2, 8]. Placing patients with ARDS in the prone position is also practiced with the recent paper by Guérin et al. (2013) showing a 16% decrease in 28-day mortality when proning ARDS patients [11]. Furthermore, practicing conservative intravenous fluid management strategies in patients who develop ARDS is frequently implemented in intensive care units. Although this conservative strategy has shown improved lung function and shortened duration of mechanical ventilation, there has been no difference in nonpulmonary organ failures and 60-day mortality [8]. Still other strategies have been individually described to help reverse this devastating syndrome but often death is still the overall outcome. Due to these high mortality rates associated with ARDS, many of the recent treatment strategies are focused on preventing the syndrome from ever developing.

There have been a number of ARDS risk-prediction models for postoperative respiratory failure described in current literature; however, many of the models are nonspecific for ARDS in critically ill surgical patients [12–15]. Those models that are more specific examine a very isolated subset of surgical patients such as cardiopulmonary bypass [16–18] or thoracic surgery [19–23]. Kor et al. described a model for predicting the risk of early ARDS based on preoperative patient characteristics [5]; however, this requires the patient to undergo surgery in order to utilize this model. Many patients in a surgical critical care unit (SICU) may not have experienced a surgical procedure or do not need surgical intervention but require mechanical ventilation due to a traumatic insult, iatrogenic injury from a bedside procedure, pathologic respiratory failure secondary to their disease process before they are able to undergo surgical intervention, and so forth.

Lung Injury Prediction Score (LIPS) is a model in which clinicians can incorporate a series of risk factors and risk modifiers to predict patients who will develop ARDS [24–27] regardless of whether or not the patient has undergone surgery (Table 1). The LIPS model allows early identification of patients at high risk for developing ARDS using routinely available clinical data even before they are admitted to the ICU [24]. Its validity and ease of use has made the LIPS model a popular choice for identifying patients at high risk for developing ARDS. Despite this, LIPS has never been validated in the critically ill surgical patient population. With a variety of models available to predict surgical patients at risk for developing ARDS based on the nature of the surgery or the requirement that the patient must have surgery we have sought to validate the LIPS model in the surgical critical care population. It would also make sense that LIPS can be used to predict mortality, yet this has never been demonstrated. We hypothesize that LIPS can be used to accurately predict surgical critical care patients at high risk for developing ARDS as well as to accurately predict mortality in this patient population.

## 2. Methods

This was an Institutional Review Board approved, prospective observational cohort study conducted at a single, tertiary Level I trauma center. The study was conducted over a six-month period from November 2012 through April 2013. It included all ventilated patients admitted to the SICU during that time. ARDS was defined using the Berlin criteria 2012 which excludes the term “acute lung injury” and classifies ARDS using the terms “mild,” “moderate,” and “severe” [28]. The diagnosis of ARDS was made by a group of three SICU physicians conducting the study, strictly using this new definition. The team caring for the patient was notified of this diagnosis (if they had not identified it on their own) and was allowed to treat the patient as they sought fit. Our SICU has already developed a low tidal volume policy, so all ventilated patients received tidal volumes between 6–8 mL/kg set forth in the Acute Respiratory Distress Syndrome Network study from 2000 [4] whether or not they already had the diagnosis of ARDS.

Lung Injury Prediction Scores were calculated for all ventilated patients only as the definition of ARDS requires the patient to receive some form of mechanical ventilation. LIPS was calculated for all ventilated patients as soon as they arrived in the SICU using the model validated by the Lung Injury Prevention Study Investigators 2011 (Table 1) [24]. Lung Injury Prediction Scores do not necessitate the patient to be mechanically ventilated; therefore, we were able to retrospectively calculate LIPS using data for the patient from the SICU admission. This is an important predictive function of LIPS as many patients in the SICU were not placed on mechanical ventilation initially but this occurred multiple days after SICU admission. Acute Respiratory Distress Syndrome diagnosis, conversely, does require the patient to be ventilated. Therefore, all ventilated patients were closely followed by the research team and determined to have developed ARDS or not during the time on the ventilator. Interestingly, the Berlin definition for ARDS does not specify how many PaO_2_ : FiO_2_ ratios must be <300 consecutively or how long the patient must be ventilated in order to use a PaO_2_ : FiO_2_ ratio <300. Therefore, if a patient had a PaO_2_ : FiO_2_ ratio <300 and met all other criteria set forth by the Berlin definition [28], that patient was determined to have ARDS in our study. Once a patient was identified as having developed ARDS via the Berlin definition, the patient remained in that cohort even though the ARDS may have resolved prior to extubation. Exclusion criteria included any patient undergoing a unilateral pneumonectomy as these patients cannot be classified under the Berlin definition of ARDS.

Baseline demographics including age, race, and gender as well as incidence of ARDS development were collected for our patient population. Primary outcomes included LIPS associated with those patients who developed ARDS using the Berlin definition. Furthermore, we sought to validate the ability of LIPS to predict mortality. Secondary outcomes included the LIPS value at which SICU patients would be deemed statistically significant for increased risk of developing ARDS.

An independent statistician, separate from the research team conducting the study, completed all data analysis. Since the primary aim of this analysis was to describe the relationship between LIPS and ARDS and between LIPS and mortality among surgical critical care patients, the distinction between continuous and categorical data was carefully described. Continuous data were described using means and standard deviations, while categorical data were described using counts and percentages. When continuous variables did not follow a normal distribution, median, minimums, and maximums were utilized instead of means and standard deviations, followed by the use of the appropriate nonparametric test. LIPS is treated as a continuous variable throughout the analysis. LIPS is compared between two-level outcome variables (ARDS and mortality) using Student's *t*-tests or Wilcoxon signed-rank tests if normality assumptions were violated. Logistic regression was used to obtain the area under the curve for LIPS predictive ability for ARDS. The SAS macro %ROCPLOT was used to obtain a ROC plot and to identify an optimal LIPS cut-off for ARDS prediction at the location of maximum sensitivity and specificity. Statistical significance was set at *p* < 0.05. All analyses were performed with SAS 9.4 (SAS Institute Inc., Cary, NC, USA).

## 3. Results

**Figure 1:**
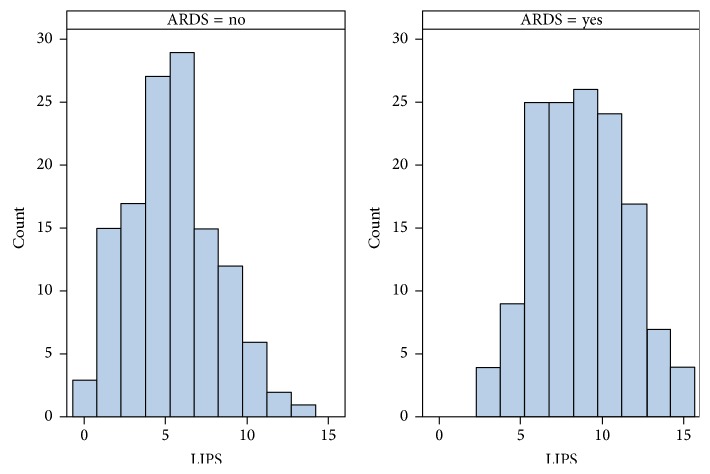
Distribution of LIPS for patients with and without the development of ARDS. Distribution of LIPS for patients who develop and do not develop ARDS. Patients with ARDS tend to have higher LIPS values.

A total of 268 patients were enrolled in the study. 155 (57.8%) patients were male (*p* = 0.738). 99 (36.9%) were African American, 147 (54.9%) were Caucasian, and 22 (8.2%) were of other race (*p* = 0.584). The average age was 57.9 ± 17.7 years. A total of 141 (52.6%) patients developed ARDS and 127 (47.4%) patients did not develop ARDS. The LIPS for patients who developed ARDS was statistically significantly higher than for those patients who did not develop ARDS.  Table 2 shows the average LIPS for a patient who developed ARDS was 8.8 ± 2.8 and the LIPS for a patient who did not develop ARDS was 5.4 ± 2.8.  Figure 1 goes on to illustrate the distribution of LIPS in patients with and without ARDS. Patients who develop ARDS tend to have higher Lung Injury Prediction Scores. Furthermore, using logistic regression modeling, for every one-unit increase in LIP score, the odds of developing ARDS increased by a factor of 1.50 (95% confidence interval: 1.34, 1.67; *p* < 0.001).

**Figure 2:**
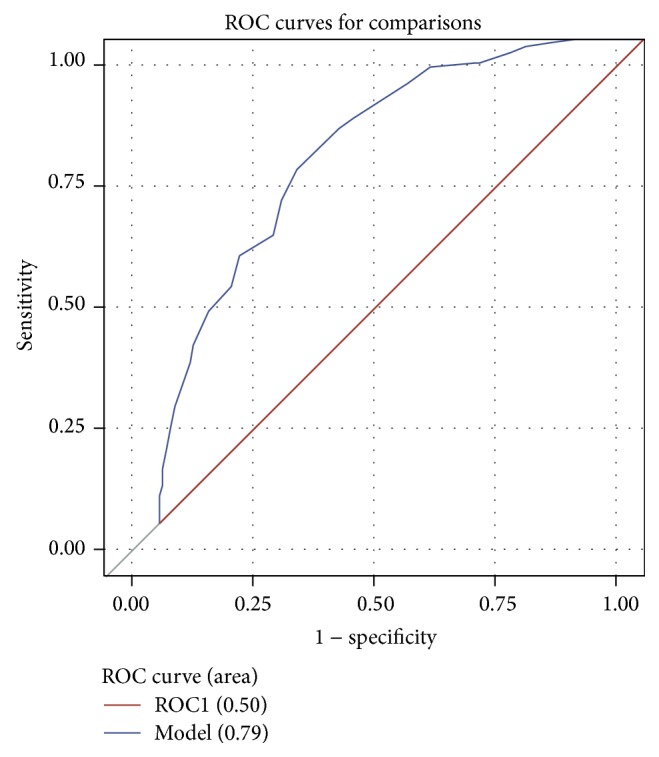
ROC curve for LIPS predicting ARDS. The area under the curve is 0.79, demonstrating LIPS is a strong predictor of the development of ARDS. Additionally, by maximizing the sensitivity and specificity, a LIPS value of 7 was determined to be statistically significant for deciding when a patient will be at high risk for developing ARDS.

Currently in the literature, a Lung Injury Prediction Score of 4 is considered the cut-off in terms of statistical significance predicting when a patient will develop ARDS [24]. This value, however, is for all ventilated critically ill patients. We were suspicious that the surgical cohort may have a different cut-off LIPS value for predicting a statistically significant risk of developing ARDS. Running the data through a ROC curve and minimizing the difference between sensitivity and specificity, we determined a LIPS value of 7 was statistically significant for predicting ARDS in the surgical critically ill patients (Figure 2). Furthermore, the area under the curve for our ROC was 0.79 (95% confidence interval: 0.74, 0.84), validating LIPS as a strong predictor of ARDS development.

The other aim of this study was to determine if LIPS can predict overall patient mortality in the surgical patient population. Utilizing a univariate analysis, for every one-unit increase in LIP score, the odds of ICU mortality, defined as death during ICU admission or within 30 days after ICU discharge, increased by 1.22 (95% confidence interval: 1.09, 1.36; *p* < 0.001) (Table 3).

## 4. Discussion

Despite changes in ventilator management as a result of the landmark ARDSnet study in 2000, ARDS remains a challenging disease process to manage in critical care settings. It is associated with increased hospital length of stay, costs, and long-term poor outcomes [29, 30]. In addition, years of clinical and bench research have not reduced the associated high mortality rate. Because of the failure of salvage therapies for diagnosed ARDS, current clinical strategies have shifted to identifying risk factors prior to lung injury development and initiating potentially preventative management options. A recent article by Ahmed et al. reported on preventable hospital events that were associated with ARDS development [31]. While some of these mentioned events could be avoided, that is, injurious tidal volumes and fluid administration, other events such as instrument failure and surgical misadventures are impossible to predict and therefore prevent.

The incidence of ARDS varies throughout the literature with rates as low as 3.6% to as high as 25% in some studies [2, 32]. Our incidence of ARDS was 52.6%, two times higher than the current quoted incidence. Although our study did not directly examine the reason for the high incidence of ARDS in our patient population, a few speculations can be proposed to explain these results. First, the definition of ARDS changed before beginning this study. By using the Berlin definition for ARDS, the disease process once known as “acute lung injury” has been replaced with “mild” ARDS. Studies prior to this change separated these two disease processes. Furthermore, there have been no studies examining the incidence of ARDS specifically in the surgical critical care patient cohort. Large intravenous fluid shifts and insults directly from the surgery or trauma may explain why the incidence of ARDS is higher in this population. There is also a certain amount of subjectivity associated with chest radiograph interpretation, which may have resulted in inclusion of patients whose clinical status did not correlate with radiologic findings. Additional studies are required to explain these findings.

LIPS has been shown to be a valid and reliable model for early prediction of high risk patients for developing ARDS, even in patients who have not yet been intubated [24–26]. A negative predictive value of 0.97 for this tool makes it a useful screening tool [24, 26]. This study further supports the use of LIPS as a trustworthy prediction model for the development of ARDS in SICU patients as demonstrated by our results. Based on our results, a Lung Injury Prediction Score of 7 or higher indicates a statistically significant increase in the risk of developing ARDS in the surgical critical care patient population. This is contrary to previous studies demonstrating the statistically significant LIPS representing an increased incidence of developing ARDS is four [24–26]. Furthermore, as the LIPS increases, the risk of developing ARDS increases. Our study statistically demonstrated that, for every one-unit increase in LIPS, the odds of developing ARDS increase by a factor of 1.50 for SICU patients.

It is important to distinguish surgical critical care patients from nonsurgical intensive care patients. Surgical critical care patients are subject to different inflammatory-provoking insults via surgery or trauma from that of medical intensive care patients, which could result in differences in the pathophysiology of ARDS. Surgical procedures and trauma expose patients to a variety of intravenous fluid shifts, blood products, and multiple medications including anesthetics and paralytics that can stress the body. The anatomical location of the surgery and the type of surgery (i.e., transplant surgery) can also result in dramatic postoperative changes in human physiology requiring specialized postoperative management. Furthermore, surgical patients tend to have large intravenous fluid shifts which can result in a higher incidence of pulmonary edema. As established from previous studies, high permeability pulmonary edema can contribute significantly to the development of ARDS [33]. The use of previously validated lung injury prediction models for surgical patients requires that patients have undergone surgery and does not include the trauma population that now accounts for a large proportion of SICU patients [5]. Our study has shown that LIPS is a valid prediction model for identifying those patients at high risk for developing ARDS regardless of the type of surgery or whether they had surgical intervention at all. LIPS also allows for the early recognition of patients at high risk for the development of ARDS. After the initial insult, ARDS can manifest in a subclinical state for approximately 30 hours before any clinical signs or symptoms are present [33]. This is wasted valuable time in which treating physicians could intervene and change management strategies to further prevent the disease process. LIPS can assist in the early recognition of surgical patients at high risk for ARDS which can provide an opportunity to change management strategies before the disease becomes too severe.

Mortality from ARDS remains high, despite improvement in new ventilator strategies [4]. This is unfortunate, as knowledge about this disease process has dramatically improved over the years. Mortality rates range anywhere from 23% [24] to as high as 68.8% [34] with most studies quoting a rate between 30 and 40 percent [35–39]. The majority of these studies were completed using the previous American-European Consensus Conference definition of ARDS [40]. Therefore, patients classified as having ARDS in past studies by enrollment criteria had PaO_2_ : FiO_2_ ≤200 mmHg [40] making it difficult to compare to this and any future study using the Berlin definition, as patients can be classified as having ARDS with a PaO_2_ : FiO_2_ ≤300 mmHg [28]. However, we did show a mortality rate of 21%, which is significantly less than the 45% mortality rate reported in the 2012 consensus statement. Reasons for these differences in mortality are outside the scope of this paper and further studies are required to answer these questions.

One of the primary outcomes of our study was to predict mortality using the LIPS model. Although previous literature has not directly looked at this relationship, it is intuitive that high LIPS correlates with a higher risk of ARDS and consequently a higher mortality rate. We found that, for every one-unit increase in LIPS, the odds of mortality in ventilated SICU patients increase by 1.22 which is statistically significant (95% confidence interval: 1.09, 1.36; *p* < 0.001). Further studies are required to validate this across all patient populations, both ventilated and nonventilated, but these initial findings show promise using this simple prediction model for quickly determining the risk of death in critical care patients.

There are several limitations to our study. First, it was an observational study from a single center. Our study compared only ventilated patients managed by a variety of critical care physicians with different ventilator management strategies. A constant management technique in our study, however, was that all patients were managed using low tidal volume strategies. A larger, prospective multi-institutional, and multicontinental double-blinded study with a ventilator management protocol is needed to better validate our results. Second, although the Berlin definition has addressed many of the limitations of the American-European Consensus Conference definition and improved the predictive validity [28], defining ARDS in the clinical setting remains dependent on some subjectivity of the diagnosing clinician, specifically through interpretation of the chest X-ray. It has been demonstrated that experts' ability to clinically separate ARDS from other heterogeneous causes of respiratory failure is limited [41, 42]. Furthermore, defining ARDS is a dynamic process and often PaO_2_ : FiO_2_ can fluctuate throughout the time the patient is on the ventilator. Further studies examining the timing and condition when to collect these values need to be established, but they are outside the scope of this paper. Finally, our study did not examine whether the early recognition of patients at increased risk of ARDS using the LIPS changed ventilator management strategies or other management strategies for that matter. Some of our intensivists may be more familiar with LIPS than others, which may have influenced ventilator management strategies.

## 5. Conclusion

Our study has shown that LIPS is a consistent and valid method for predicting the development of ARDS and mortality in ventilated surgical critical care patients. Unlike previously described models to predict ARDS in surgical patients, LIPS is easy to calculate and does not require the patient to have undergone surgery or even be mechanically ventilated initially. Furthermore, our study suggests a LIPS of 7 (not 4) as the statistically significant transition point for considering surgical critical care patients at high risk for developing ARDS. Although further studies are required to validate our results, we recommend clinicians treating in the SICU utilize the LIPS model to predict patients at higher risk for ARDS. By identifying these patients early, changes in ventilator management strategies, fluid management strategies, and so forth may be implemented to reduce the risk of developing ARDS and help reduce overall mortality.

## Figures and Tables

**Table 1:** Lung Injury Prediction Score calculation worksheet (with permission from the American Thoracic Society) [24].

	LIPS points	Examples
Predisposing conditions		(1) Patient with history of alcohol abuse with septic shock from pneumonia requiring FI O_2_ > 0.35 Emergency room: sepsis + shock + pneumonia + alcohol abuse + FIO_2_ > 0.35 1 + 2 + 1.5 + 1 + 2 = 7.5 (2) Motor vehicle accident with traumatic brain injury, lung contusion, and shock requiring FIO_2_ > 0.35 Traumatic brain injury + lung contusion + shock + FIO_2_ > 0.35 2 + 1.5 + 2 + 2 = 7.5 (3) Patient with history of diabetes mellitus and urosepsis with shock sepsis + shock + diabetes 1 + 2 − 1 = 2
Shock	2	
Aspiration	2	
Sepsis	1	
Pneumonia	1.5	
High-risk surgery^*∗*^		
Orthopedic spine	1	
Acute abdomen	2	
Cardiac	2.5	
Aortic vascular	3.5	
High-risk trauma		
Traumatic brain injury	2	
Smoke inhalation	2	
Near drowning	2	
Lung contusion	1.5	
Multiple fractures	1.5	
Risk modifiers		
Alcohol abuse	1	
Obesity (BMI > 30)	1	
Hypoalbuminemia	1	
Chemotherapy	1	
FIO_2_ > 0.35 (>4 L/min)	2	
Tachypnea (RR > 30)	1.5	
SpO_2_ < 95%	1	
Acidosis (pH < 7.35)	1.5	
Diabetes mellitus^*∗∗*^	−1	

BMI = body mass index; RR = respiratory rate; SpO_2_ = oxygen saturation by pulse oximetry.

^*∗*^Add 1.5 points in case of emergency surgery.

^*∗∗*^Only in cases of sepsis.

**Table 2:** Average LIPS for patients with and without the development of ARDS.

		No ARDS	ARDS	*p* value
LIPS	Mean (SD)	5.4 (2.8)	8.8 (2.8)	<0.001^*∗∗*^
	Median (min, max)	5.5 (0, 13)	8.5 (2.5, 15)	

Univariate relationship between LIPS and ARDS status. LIPS is statistically higher in patients who develop ARDS as compared to those who do not. ^*∗∗*^Statistical significance.

**Table 3:** Univariate association between LIPS and mortality.

	LIPS mean (SD)	LIPS median (min, max)	OR (95% CI)	*p* value
Alive (*N* = 208)	6.8 (3.3)	6.5 (0, 15)	(Reference)	0.002^*∗∗*^
Deceased (*N* = 32)	8.8 (2.5)	9 (4, 13)	1.22 (1.08, 1.38)	

Using those patients who survived as the reference, LIPS significantly predicts mortality as every 1-unit increase in LIPS raises the odds of mortality by 1.22. ^*∗∗*^Statistical significance.
